# Efficient OPSL-pumped mode-locked Yb:Lu_2_O_3_ laser with 67% optical-to-optical efficiency

**DOI:** 10.1038/srep19090

**Published:** 2016-01-07

**Authors:** Alexander M. Heuer, Clara J. Saraceno, Kolja Beil, Günter Huber, Christian Kränkel

**Affiliations:** 1 Institut für Laser-Physik, Universität Hamburg, 22761 Hamburg, Germany; 2 The Hamburg Centre for Ultrafast Imaging, Universität Hamburg, Hamburg 22761, Germany; 3 Institute for Quantum Electronics, ETH Zurich, 8093 Zurich, Switzerland

## Abstract

We present a mode-locked Yb:Lu_2_O_3_ laser with up to 67% of optical-to-optical efficiency. By utilizing a high brightness optically pumped semiconductor laser (OPSL) as a pump source and using a semiconductor saturable absorber mirror (SESAM) we obtained self-starting mode locking. A pulse duration of 571 fs at 4.73 W of average output power with an optical-to-optical efficiency of 67% was achieved. In a slightly different cavity configuration the pulse duration was reduced to 313 fs at 2.16 W of average output power. In both cases the pulse duration was longer than the Fourier limit and the spectrum supports significantly shorter pulse durations. The laser wavelength is centered at 1034 nm and the repetition rate is 100.76 MHz in both cases. In continuous wave fundamental mode operation the optical-to-optical efficiency was as high as 78% with output powers exceeding 5 W.

Yb^3+^-doped materials are of great interest for the generation of ultra-short pulses via semiconductor saturable absorber mirror (SESAM)^1^ and Kerr-lens^2^ mode locking. The simple energy level scheme of Yb^3+^ prevents undesirable effects like excited-state absorption or cross relaxation and its Stokes efficiency exceeding 90% allows for very efficient laser operation^3^. Yb^3+^-doped materials have demonstrated their potential for ultra-short pulse generation via mode locking. The shortest pulses achieved with any Yb^3+^-doped material so far were generated by using a high brightness fiber laser pump source. In this case the pulse duration was 32 fs using Yb:CaGdAlO_4_ as the gain medium^4^. At 90 mW of average output power and 3.6 W of pump power the optical-to-optical efficiency amounted to 2.5%. This concept is also suitable for further scaling of the average output power^5^. Nevertheless, the range of possible emission wavelengths of Yb:silicate fibers operated at their zero-phonon-line is limited to the narrow peak around 976 nm. On the other hand, power scaling of mode-locked oscillators using Yb:YAG in the thin disk laser geometry^6^ up to now resulted in more than 250 W of average output power^7^,^8^ and pulse energies approaching 100 μJ^9^ at sub-ps pulse durations. The cubic sesquioxides yttria (Y_2_O_3_), lutetia (Lu_2_O_3_), and scandia (Sc_2_O_3_) are favourable host materials for efficient, high power Yb^3+^-doped ultra-fast lasers. They combine excellent thermomechanical properties and broad spectroscopic features^10^. In particular, Yb:Lu_2_O_3_ has been shown to be suitable for high power laser operation as well as the generation of ultrashort pulses^11^,^12^. Several hundred watts of continuous wave (cw) output power with optical-to-optical efficiencies exceeding 70% have been achieved with this material in the thin disk laser geometry^13^,^14^. In bulk geometry, mode-locked pulses as short as 71 fs at an average output power of 1.1 W were realized using Yb:Lu_2_O_3_^15^. The optical-to-optical efficiency in this case was 16%. Even shorter pulses were obtained using Yb:Sc_2_O_3_ and Yb:Y_2_O_3_ simultaneously in one cavity. In this case, 53 fs at 1 W of average output power and 19% optical-to-optical efficiency were achieved^16^. In high average power mode-locked operation in the thin disk laser geometry, Yb:Lu_2_O_3_ previously allowed for very high optical-to-optical efficiency of up to 43% at 141 W of average output power in 738 fs pulses^17^. However, combining ultra-short pulse generation, high optical-to-optical efficiency and multi-watt power operation is challenging. One of the reasons is that stable mode locking requires fundamental transversal mode operation which can not be efficiently realized without a perfect overlap between pump and laser mode. Using a pump source with diffraction-limited beam quality is beneficial in this regard. Ti:sapphire lasers are widely used laboratory pump sources for this purpose, but their output power is typically limited to a few watts in the wavelength range between 940 nm and 990 nm required for pumping Yb^3+^-doped laser materials^18^. In contrast, optically pumped semiconductor lasers (OPSL)^19^ can deliver several tens of watts of output power in diffraction-limited beam quality^20^,^21^ at higher efficiencies and in a less complicated setup compared to Ti:sapphire lasers (see Fig. 1a), while providing a decent wavelength tuning range that allows to address the zero-phonon-line absorption peak of most Yb-doped materials (see Fig. 1b). As such, OPSLs are regarded as ideal pump sources for highly efficient cw bulk and waveguide lasers as well as ultra-fast Yb-based laser oscillators^22^,^23^. Here we report on the first OPSL-pumped mode-locked Yb-doped bulk laser with unprecedented efficiency in cw and pulsed operation. Using Yb:Lu_2_O_3_ as the gain material we achieved pulse durations as short as 313 fs with several watts of average output power at optical-to-optical efficiencies of up to 67%. This is, to the best of our knowledge, the highest optical-to-optical efficiency reported for any passively mode-locked laser oscillator. The mode locking is self-starting and induced using a semiconductor saturable absorber mirror (SESAM). In cw fundamental mode operation the setup allowed for slope efficiencies of up to 86% with more than 5 W of output power.

## Results

### Experimental Setup

**Figure 1:**
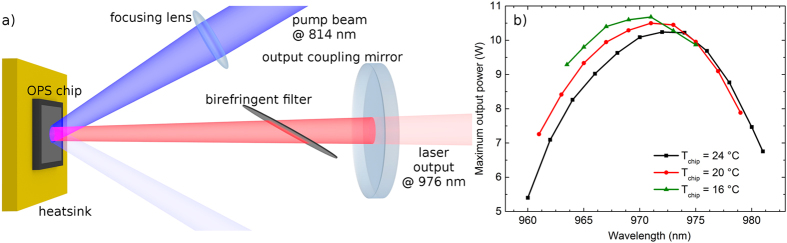
Schematic of a tunable OPSL setup. The high absorption of the InGaAs gain chip supplied by Coherent Inc. allows for >99% absorption in a single pass (**a**). Tuning range and wavelength-dependent maximum output power of the OPSL for three different OPS chip temperature settings T_chip_ (**b**). The laser emission bandwidth was below 0.5 nm and the M^2^ was less than 1.2. The pump power amounted to approximately 30 W.

**Figure 2:**
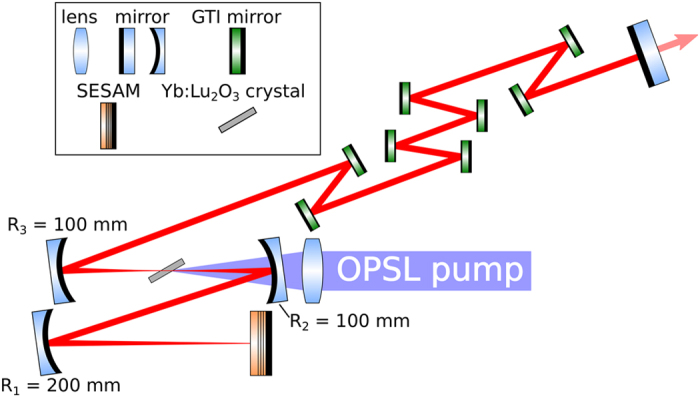
Experimental setup of the mode-locked OPSL pumped Yb:Lu_2_O_3_ oscillator.

**Figure 3:**
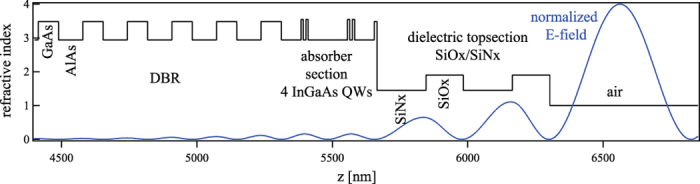
Design schematic of the SESAM used in the modelocking experiments.

The laser experiments were performed in a ≈1.5 m long fundamental mode cavity formed by three concave mirrors with radii of R_1_ = 200 mm and R_2,3_ = 100 mm, and up to 8 plane GTI mirrors with a group delay dispersion (GDD) of −550 fs^2^ each. A SESAM and a plane output coupling mirror served as the end-mirrors of the cavity (see Fig. 2). Depending on the alignment of the resonator and the distance between the mirror R_1_ and the SESAM, the spot-diameter on the SESAM could be varied between 60 μm and 120 μm. The 2 mm thick Yb(3%):Lu_2_O_3_ crystal was grown in our laboratory using the heat exchanger method (HEM)^11^,^24^. It is mounted at Brewster’s angle in a watercooled copper heatsink. This increases the effective length of the gain material to 2.25 mm. The SESAM was grown in the FIRST cleanroom facilities at the ETH Zurich. It features a modulation depth of 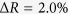, non-saturable losses of 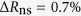, a saturation fluence of 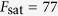
μJ/cm^2^, and a 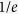 recovery time of 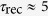
ps. The SESAM was designed for high-power operation and short recovery time using the guidelines shown by Saraceno *et al.*^25^. The absorber section, grown on a standard AlAs/GaAs distributed Bragg reflector, consists of 4 quantum wells placed two by two in consecutive antinodes of the electric field standing wave pattern. A dielectric top section consisting of two pairs of SiN_x_/SiO_x_ was applied to increase the saturation fluence and damage threshold, and reduce the non-saturable loss of the sample (see Fig. 3). The average power of the mode-locked laser was measured with a Coherent PowerMax USB powermeter. To record the spectrum of the pulses we used a Yokogawa AQ6370C optical spectrum analyzer with a wavelength resolution of 0.02 nm. The pulse duration was determined by a Femtochrome FR-103XL autocorrelator and recorded using a Tektronix DPO 4104B 1 GHz oscilloscope. Finally, the repetition rate was detected by an Agilent N9320B RF analyzer. The OPSL has been provided by Coherent Inc. and is capable of delivering up to 10 W of output power at near diffraction-limited beam quality. It features an InGaAs gain chip and is pumped by an 814 fiber-coupled laser diode and tunable from 960 nm to 982 nm by means of an intracavity birefringent filter (see Fig. 1a). For pumping the Yb(3%):Lu_2_O_3_ crystal we tuned the emission wavelength of the OPSL to the zero-phonon-line absorption at 976 nm. The FWHM spectral bandwidth of the OPSL was below 0.5 nm. After passing a Faraday rotator, several deflection mirrors, and the incoupling mirror we were left with 7.4 W of diffraction-limited pump power incident on the laser crystal. The pump light was focused into the laser crystal through a curved mirror with a radius of curvature of R_2_ = 100 mm by means of a lens with a focal length of 65 mm. This results in a beam waist diameter of about 65 μm in the crystal.

### Experimental Results

**Figure 4:**
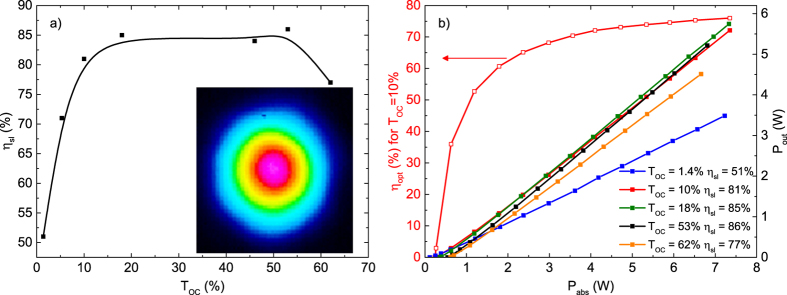
SSlope efficiencies *η*_sl_ of OPSL-pumped Yb:Lu_2_O_3_ vs. output coupling transmissions T_OC_ and transversal mode profile at maximum cw output power (**a**). lope efficiencies 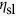 of the cw Yb:Lu_2_O_3_ laser (**b**) at different output coupling transmissions and optical-to-optical efficiency 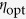 at 10% output coupling transmission vs. absorbed pump power P_abs_.

**Figure 5:**
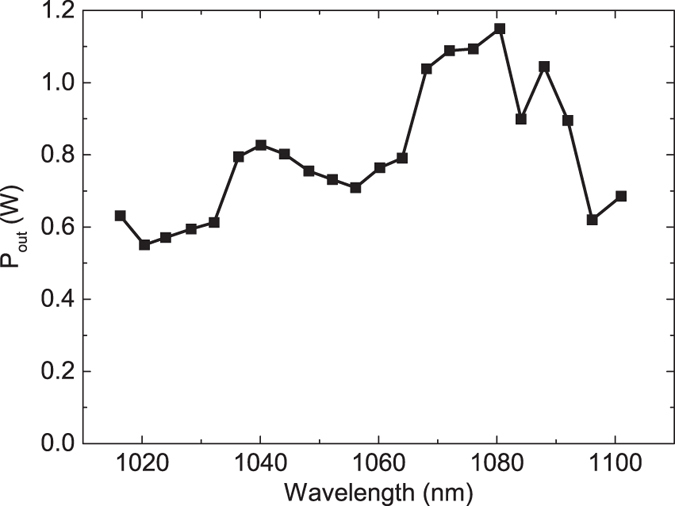
Output power through a 0.4% output coupling mirror vs. emission wavelength of an OPSL-pumped Yb:Lu_2_O_3_ laser in cw operation at an absorbed pump power of 5 W. The wavelength was tuned with a 1.5 mm birefringent filter.

**Figure 6:**
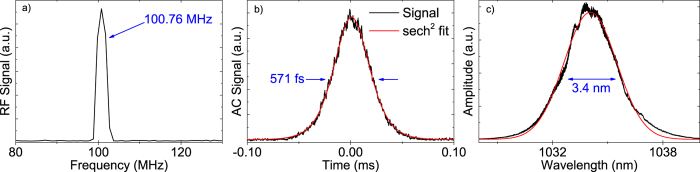
Repetition rate (**a**), autocorrelator signal (**b**), and wavelength spectrum (**c**) at the highest average output power of 4.74 W.

First we characterized the laser performance in a fundamental mode resonator under OPSL-pumping. For this purpose we replaced only the SESAM with a highly reflective (HR) mirror, while the GTI mirrors remained in the cavity. Due to the effective gain medium thickness of 2.25 mm in Brewster’s angle the absorption efficiency was at least 96% in all cases. We obtained slope efficiencies 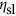 exceeding 70% for all output coupler transmissions between 5% and 62%. The highest slope efficiency of 86% was observed at a high output coupler transmission of 53% (cf. Fig. 4a). In this case the threshold was as high as 0.56 W of absorbed pump power. At lower output coupler transmissions of 10% the laser threshold was reduced to 0.24 W of absorbed pump power. In this case the slope efficiency was still as high as 81% and the optical-to-optical efficiency 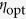 with respect to the absorbed pump power amounted to 78% (see Fig. 4b). Even with respect to the incident pump power the optical-to-optical efficiency was as high as 75%. In all experiments the laser operated in TEM_00_ mode at a wavelength of 1034 nm with a beam quality of M^2^ < 1.1. The mode profile of the laser at maximum output power can be found in the inset of Fig. 4a. The maximum output power was 5.75 W at an absorbed pump power of 7.33 W using an output coupling mirror with a transmission of 18% for the laser wavelength. In addition we performed tuning experiments using a birefringent filter with a thickness of 1.5 mm under Brewster’s angle inside the cavity. At a low output coupler transmission of 0.4% we achieved continuous wavelength tuning between 1016 nm and 1101 nm while maintaining watt-level output power (see Fig. 5). The tuning range was limited by the free spectral range of the filter. Due to the high gain of the laser crystal the tuning curve is very smooth and did not show a strong dependence on the output wavelength. The cavity was then set up for mode-locking by replacing the highly reflective end mirror with the SESAM. Using a wedged output coupling mirror with a transmission of 10% for the laser wavelength, we obtained similar laser efficiencies in mode-locked operation as in the cw experiments. This confirms the low non-saturable losses of the SESAM sample. In a first experiment targeting high average power we obtained stable mode locking introducing a total of −6600 fs^2^ GDD per cavity round trip spread across 6 GTI mirrors. In this configuration the laser threshold was reached at about 0.3 W of pump power. Stable mode locking was obtained for absorbed pump powers between 0.7 W and the maximum available value of 7.1 W. At the highest pump power we achieved a pulse duration of 571 fs at an average output power of 4.74 W. At a repetition rate of 100.76 MHz the pulse energy amounted to 47 nJ (see Fig. 6). This corresponds to an optical-to-optical efficiency of 67% with respect to the absorbed pump power and 65% with respect to the incident pump power. These are, to the best of our knowledge, the highest optical-to-optical efficiencies achieved from any mode-locked laser oscillator. The pulses had a spectral bandwidth of 3.4 nm (FWHM) corresponding to a time-bandwidth-product of 0.527 which is 1.67 times above the Fourier limit for sech^2^-pulses of 0.315. In further experiments we aimed for shorter pulse durations. It should be noted that unlike the case of dispersion compensation via prisms^26^,^27^, GTI mirrors do not allow for a continuous tuning of the cavity dispersion to the optimum value. Moreover, to retain a high efficiency we avoided using a Brewster plate typically applied in mode-locked thin-disk lasers for self-phase modulation tuning^28^. Nevertheless, the self-phase modulation (SPM) mostly generated in the laser crystal can be tuned to a certain extent by changing the pump power and thus the average intracavity laser power. We also modified the SPM by increasing the intensity of the laser mode in the crystal. For this purpose we decreased the beam waist in the crystal by increasing the distance between the mirrors R_2_ and R_3_. At the same time we decreased the distance between R_1_ and the SESAM. In this way we achieved the shortest pulse durations. At an absorbed pump power of 3.36 W we obtained 313 fs pulses at an average output power of 2.16 W and a slightly reduced optical-to-optical efficiency of 64%. In this case the optical spectrum had a bandwidth of 6.2 nm resulting in a time-bandwidth-product of 0.545, about 1.7 times above the fourier limit. Adding further GTI mirrors with lower individual GDD values could be beneficial to further shorten the pulse duration in future experiments.

*Tokurakawa et al.* demonstrated, that dispersion compensation with prisms allows for pulse durations as short as 71 fs with Yb:Lu_2_O_3_ in a similar cavity, which was however operated in the Kerr-lens mode-locked regime^15^. In this case the optical-to-optical efficiency was reduced to 16%. Therefore, despite the significantly shorter pulse durations of *Tokurakawa et al.* our peak power levels are comparable.

## Discussion

In conclusion we have presented an ultra-high efficiency ultra-short pulse mode-locked laser based on Yb(3%):Lu_2_O_3_. Using an OPSL as a pump source we achieved fundamental mode laser operation at 78% of optical-to-optical efficiency in the cw regime. In mode-locked operation the pulse duration was 571 fs at the highest average output power of 4.74 W, only limited by the available pump power. After realignment of the cavity shorter pulses of 313 fs duration were obtained at an average output power of 2.16 W. In both cases the pulse duration was longer than the Fourier limit and the spectrum of the shortest pulses supports durations below 200 fs. In these experiments, the optical-to-optical efficiency reached 67% and 64%, respectively. To the best of our knowledge, these are the highest optical-to-optical efficiencies obtained with any mode-locked laser oscillator. These efficiencies are significantly higher than reported in previous work with Yb^3+^-doped sesquioxides^29^ or any other modelocked laser^30^,^31^. Further improvement in terms of pulse duration can be expected from a refined cavity dispersion management, as Yb:Lu_2_O_3_ has been shown to support sub-100 fs pulse durations^15^.

These results emphasize the significance of OPSLs as a pump source especially in laboratory environments. We regard the unique combination of high output power and excellent beam quality^21^, while still offering a decent amount of wavelength tunability, as extremely beneficial for this field of research.

## Additional Information

**How to cite this article**: Heuer, A. M. *et al.* Efficient OPSL-pumped mode-locked Yb:Lu_2_O_3_ laser with 67% optical-to-optical efficiency. *Sci. Rep.* **6**, 19090; doi: 10.1038/srep19090 (2016).
